# Ring-Split: Deadlock-Free Routing Algorithm for Circulant Networks-on-Chip

**DOI:** 10.3390/mi14010141

**Published:** 2023-01-05

**Authors:** Aleksandr Y. Romanov, Nikolay M. Myachin, Evgenii V. Lezhnev, Alexander D. Ivannikov, Ahmed El-Mesady

**Affiliations:** 1 HSE University, Moscow 101000, Russia; 2 OOO “OZON TEKHNOLOGII”, Moscow 123112, Russia; 3 Institute for Design Problems in Microelectronics of Russian Academy of Sciences, Moscow 124365, Russia; 4 Department of Physics and Engineering Mathematics, Faculty of Electronic Engineering, Menoufia University, Menouf 32952, Egypt

**Keywords:** network-on-chip, routing algorithm, deadlock, circulant topology, NoC modeling

## Abstract

This article considers the usage of circulant topologies as a promising deadlock-free topology for networks-on-chip (NoCs). A new high-level model, Newxim, for the exploration of NoCs with any topology is presented. Two methods for solving the problem of cyclic dependencies in circulant topologies, which limit their applications for NoCs due to the increased possibility of deadlocks, are proposed. The first method of dealing with deadlocks is universal and applicable to any topology; it is based on the idea of bypassing blocked sections of the network on an acyclic subnetwork. The second method—Ring-Split—takes into account the features of circulant topologies. The results of high-level modeling and comparison of the peak throughput of NoCs for circulant and mesh topologies using deadlock-free routing algorithms are presented. It was shown that a new approach for routing in circulants (compared to mesh topology) shows up to 59% better network throughput with a uniform distribution of network load.

## 1. Introduction

The constant development of technologies and the increase in the number of possible placements of computing nodes on a single chip pose increasingly tough challenges for the communication subsystem of Networks-on-Chip (NoCs). In addition to the structure of routers, the NoC characteristics are significantly affected by the network topology, the method of data transfer control, and the routing algorithm [1]. An important direction for the NoC design is the use of circular topologies. So, Spidergon topology is used in STNoC software developed by ST Microelectronix [2,3]; there are examples of the usage of the Octagon topology [4] and its 3D version Octagon for Ubiquitous Computing (OUC) [5], Midimew [6], etc. All of them are related to circulant topologies whose optimal configurations in the context of the application as a topological basis in the design of NoCs are still little studied.

**Figure 1:**
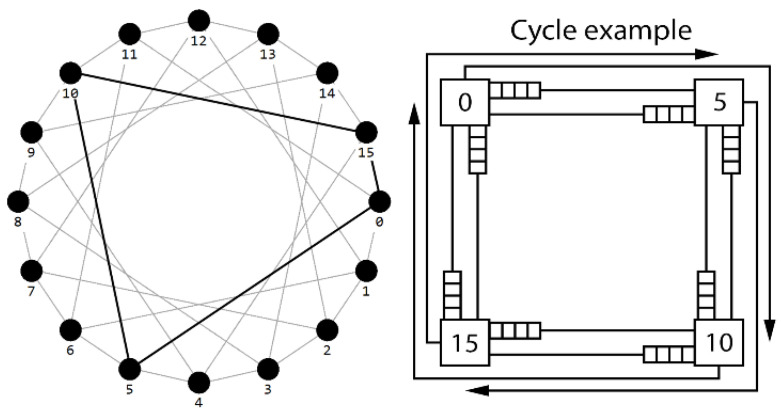
Circulant topology C (16; 1, 5). 0−5−10−15−0, 0−5−6−11−0, etc.—cycles in the topology.

Circulant topologies [7] (Figure 1) have better characteristics compared to mesh and torus topologies [8]. They have already been widely used in various networks: they are used as a topology of local computer networks [7,9,10,11], data center networks [12], high-performance computing clusters [13,14,15], communication networks [16], etc. All this suggests that circulants are promising topologies for the NoC design. Wherein the usage of new topologies in NoCs requires the development of routing algorithms to not only build packet propagation paths but also guarantee the absence of deadlocks resulting in a complete halt in data transmission through the network. Work [17] presents some routing algorithms in circulant topologies with different efficiency and implementation complexity. The most complex Pair Exchange Algorithm allows calculating the route of a packet in the form of the number of necessary transitions of the packet along the generators of the circulant when it is sent. Routing algorithms for optimal circulants are easier to implement, but they are not suitable for ring-type circulants. A simpler implementation, the clockwise routing algorithm developed for use in ring-type circulants, does not guarantee optimal routes. In addition to the routing algorithms mentioned in [17], there are many different implementations of routing algorithms developed at different times by a number of authors for different classes of circulant networks [18,19,20,21,22,23,24]. The existing algorithms for routing in circulant topologies have both advantages and disadvantages and can be used depending on the task. But they have a common critical drawback, which is that all these algorithms do not guarantee the absence of deadlocks in data transfer in the real on-chip network. The problem of deadlock prevention in NoCs has been well studied in relation to such topologies as mesh and torus, which has led to the emergence of a whole class of algorithms designed to deal with deadlocks [25]. Since circulants are regular topologies with a symmetrical structure, the occurrence of deadlocks in them takes place due to the overlap of the paths provided by existing routing algorithms (Figure 1).

Store-and-forward deadlock (hereinafter referred to as simply deadlock) is a cyclic dependence of several packets, which leads to mutual blocking of their further movement through the network. A sufficient condition for the occurrence of deadlocks is the existence of “nodal cycles” [26] formed by the imposition of possible routes. Such algorithms as XY [27,28], Odd-Even [29], SOE [30], Duato’s protocol [31], and others [25] for mesh topology, by using simple rules, guarantee the existence of many possible paths that do not have overlaps, and this ensures the absence of deadlocks and significantly improves the network performance. The importance of providing deadlock-freedom for the routing algorithms in NoCs is confirmed by the fact that hundreds of variations have already been developed for various topologies and NoCs implementations, which is reflected in a number of surveys [9,32,33,34]. Meanwhile, the use of circulant topologies in NoCs has still been little studied (although reflected in some works) [8,17,35].

The problem of ensuring deadlock-freedom in such topologies has not been researched. As for the circulant topologies, they contain a large number of cycles and have a complex structure, which makes it difficult to develop algorithms that provide many paths satisfying the “nodal cycles” constraint. The simplest example of a “nodal cycle” is shown in Figure 1, where each of the routes has a common transition with two other routes, which leads to the possibility of deadlocks.

Thus, the purpose of this work is to develop and test deadlock prevention algorithms in NoCs with circulant topologies. This article proposes a new method of dealing with deadlocks—Ring Split (RS)—and the implementation of the Acyclic Subnetwork Method (ASM) for routing, solving the problem of deadlocks in NoCs with circulant topologies and allowing the use of existing routing algorithms (Section 3, Section 6 and Section 8).

To evaluate the effectiveness of the proposed solutions, it was necessary to conduct high-level NoC modeling. There are a large number of software simulators [36,37] developed to solve this problem for various topologies. But the idea of using circulant topologies in the NoC design is quite new, and none of the existing simulators provides the necessary tools for this study. For this reason, a high-level model based on the Noxim simulator [38] was developed (Section 2). The simulation results are given in Section 6 and Section 7.

## 2. Newxim Model for Studying NoCs with Circulant Topologies

To create our own high-level NoC model, we used the Noxim simulator [38]. This model is an open-source software hosted on Github. The simulator was developed at the University of Catania using the SystemC language. One of its main tasks is NoC modeling with the use of wireless data transmission channels; it was used by its authors to demonstrate the effectiveness of the proposed technical solutions for wireless data transmission in NoCs [39]. At the same time, it is good for modeling ordinary networks, such as mesh, butterfly, omega, etc. Noxim has the ability to flexibly configure simulation parameters. It is possible to set NoC power consumption parameters, buffer size, number of virtual channels, routing algorithm, clock rate, packet size, and much more.

In the basic implementation, the Noxim simulator does not support arbitrary topologies and, accordingly, routing algorithms in them, as well as specialized traffic control methods, which significantly limits its capabilities. Thus, the simulator was significantly redesigned and expanded with additional functionality. At present, the simulator has already been changed by more than 70%, and therefore, it is already being developed as a separate project called Newxim [40]. It contains very detailed documentation, which reflects its benefits, possibilities for use, and step-by-step instructions.

**Figure 2:**
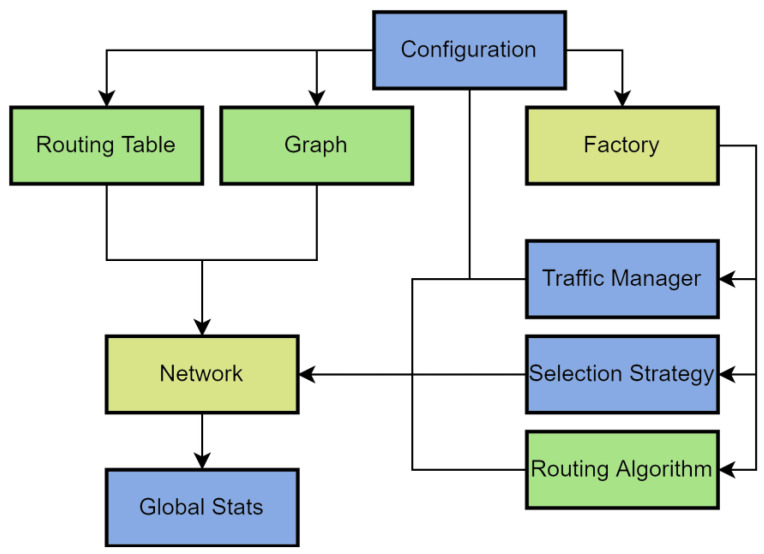
Structure of the Newxim simulator.

The implementation of support for arbitrary topologies by the simulator required changes in its original structure. Figure 2 shows the general structure of the simulator; the colors show how much the modules were changed compared to the original model (green—more than 90%, blue—more than 60%, yellow—more than 30%).

The configurator takes the input parameters (that describe the future modeling process), checks the validity of the values, and builds the network structure. The description of the network structure is passed to the simulator core, which creates the network itself and starts the simulation. Next, the metrics of the simulation cycles performed are calculated and based on the obtained values, and the final report is generated.

The network is created on the basis of a graph, set either manually or according to one of the built-in templates, which allows the modeling of arbitrary topologies. The routing algorithm for the topology is specified using the routing table. To provide the required flexibility of the model, it was necessary to change the implementation of routers to support an arbitrary number of ports.

The network generation process was also amended. Since some traffic control methods require the model to have virtual channels [25], it was decided to implement virtual channel simulation by introducing additional links between pairs of routers [41]. The disadvantage of this method is that additional links increase the estimated network throughput at the expense of hardware costs. This can lead to some bias when comparing a network using additional channels with a network that does not use them, which must be taken into account when analyzing the simulation results.

The simulator was further extended by the Newxim Manager multi-simulation management system. It supports multiple runs of simulations based on predefined parameters. The configuration of both general parameters and specific parameters for each series is supported. A series is a collection of simulations of the same type, depending on a variable argument, whose range and step are set before starting.

The program includes a flow control system, so it is possible to run an arbitrary number of simulations in parallel. The distribution of threads occurs automatically; the user sets only the size of the thread pool. It is not recommended to set the thread pool larger than the processor (on which the simulation is being performed) can physically execute. Although the program continues to work correctly, this negatively affects the simulation time.

To visualize topologies and routing algorithms, the Simple Routing Analyzer module was added to the simulator. The tool has a graphical interface and supports setting arbitrary topologies through an adjacency matrix (Figure 1). Also, there are built-in matrix generators for networks with mesh topology and circulant. The program supports various routing algorithms introduced during the research process. And in addition, there is a load testing module with sequential failure of nodes and collection of the metrics of this process.

For an objective analysis of the developed routing algorithm, it is necessary to compare it with existing analogs. Despite the possibility of modifying the simulator to add new algorithms, it was necessary to provide a large library of standard implementations. For this purpose, a large number of routing algorithms are built into the simulator. Routing algorithms for mesh networks: XY, West First, O1TURN, XY-YX, Negative First, North Last, and Odd-Even [25,27,28,29,30]. The CLUE algorithm [42,43] is implemented for networks with a torus topology. Also, during the development process, versions of algorithms for networks with a dedicated subnetwork to bypass deadlock situations were created: Virtual Subnetwork and Fixed Subnetwork.

The routing algorithm functions in conjunction with the selection strategy. It is responsible for the way in which one path is determined from the set provided by the algorithm. The simulator has a set of built-in selection strategies: Random, Buffer Level, Keep Space, Random Keep Space, RS, and Virtual RS.

## 3. Description of the Ring-Split Algorithm

Since deadlocks occur as a result of the overlap of possible packet routes in “nodal cycles” [26] (Figure 1), it is necessary to discard some of the possible routes in order to break these cycles. At the same time, the breaking of cycles in the graph also leads to the breaking of all “nodal cycles” lying on it.

Let there be an undirected circulant $C\left(N;s_{1},s_{2},\ldots ,s_{k}\right)$, where N is the number of nodes, k is the dimension of the graph, and $s_{i},\;1\leq i\leq k$ are the generators of the graph [7]. Such a graph can be divided into k subgraphs constructed from edges corresponding to generators and vertices incident to them. Each subgraph consists of $m_{j}\geq 1$ disconnected cyclic graphs, where $m_{j}=GCD\left(N,s_{i}\right)$.

Let’s call each of the subgraphs the level $L_{j}$. All edges belonging to the corresponding level will be called $l_{ji}\in L_{j}$. Cyclic graphs, corresponding to each level, are called rings R(j,r), where j is the number of the level to which the ring belongs, r is the number of the corresponding cyclic graph. Thus, we obtain the set of all possible cycles for each level, consisting of all the rings belonging to it.

Let us divide all possible cycles in a circulant into two groups: those that lie directly on the levels, $L_{j,}$ and all other cycles of the circulant formed by the initial connectivity of the levels. By introducing a restriction on the transition between edges belonging to different levels, we can break all the cycles of the second group. Let us write down the condition under which the transition from the edge $l_{si}\in L_{s}$ to the edge $l_{di}\in L_{d}$ is allowed. The transition between levels is possible only down their hierarchy, that is, if the condition s≥d. Thus, a system of rules is obtained in which the occurrence of “nodal cycles” is possible only within the framework of the existing levels $L_{i}$. That is, the task is reduced to organizing deadlock-free routing at separate levels, each of which consists of $m_{j}$ disconnected cyclic graphs.

## 4. Application of the Ring-Split Method to the Routing Algorithm

Each route from A to B of the circulant $C\left(N;s_{1},s_{2},\ldots ,s_{k}\right)$ can be represented as a k-dimensional vector $\left\{x_{1},x_{2},\ldots ,x_{k}\right\}$, where $x_{i}\in Z$—number of clockwise moves along the i-th generator ($x_{i}>0$) or counterclockwise moves ($x_{i}<0$) required to reach node B, starting from node A. Thus, the condition necessary for the implementation of the RS algorithm is that moving along $x_{i},\;i=\left(\bar{2,k}\right)$ should be carried out earlier than along $x_{i-1}$. As a result, packets will pass through all the generators of the route in a strictly defined order.

**Figure 3:**
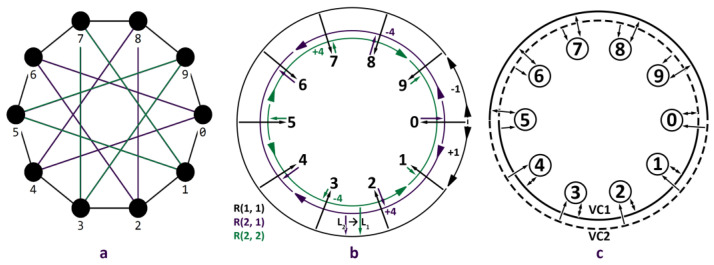
Structure of levels of the circulant C(10;1, 4): (**a**) graph scheme; (**b**) levels of the graph; (**c**) virtual channels scheme.

Figure 3a,b shows the division into levels and possible movements of packets for the circulant C(10; 1, 4). The packet, going from node A to node B, enters one of the rings R(2,1), R(2,2) and performs, according to the priority, all the necessary $x_{2}$ movements, after which it goes to the ring R(1,1), where it makes the remaining $x_{1}$ moves and reaches node B. With this scheme, the packet can only move from level $L_{2}$ to level $L_{2}$ or $L_{1}$, which satisfies the condition $l_{si}\in L_{s},l_{di}\in L_{d},s\geq d$.

## 5. Organization of Routing in Rings

The proposed restrictions on packet movements are universal and do not depend on the selected routing algorithm type. In this case, the type of routing strongly affects the routing within each individual ring R(j,r). Consider the possible types of routing that can be used in conjunction with the presented system of restrictions to eliminate deadlocks.

### 5.1. Store-and-Forward Routing

Store-and-forward [25] is the simplest type of routing to implement. In order to guarantee the absence of deadlocks within each ring, it is necessary to transmit those packets that lie in the buffers belonging to the corresponding ring. Thus, for level $L_{1}$, the movement of all packets in both directions will be guaranteed, and for level $L_{2}$, the movement of all packets will be guaranteed, except for those that are in the queue for transition to level $L_{1}$ or blocked by them. This can be implemented by fixing the order in which packets are arbitrated in the buffers. The decision order for packets corresponds to the order of the levels to which the respective packet buffers belong. The order within one level can be arbitrary. The decision to transmit packets belonging to local buffers is made last.

### 5.2. Wormhole Routing

The implementation of wormhole routing [25] has significant limitations due to the fact that the tails of some packets can prevent the continuous movement of others. To solve this problem, it is proposed to divide each ring R(j,r) of the circulant into two virtual channels. Since the packet will make no more than ⌊N/2⌋ hops in the process of moving around the ring (by dividing the packets arriving in different halves of the ring through different virtual channels), it is possible to guarantee the absence of blocking and a uniform load on each of the channels. Thus, in accordance with Figure 3c, packets entering the ring from the first half of the nodes in the cycle are sent to VC1, while packets from the second half will move through VC2. In this case, the packet can leave the ring at any time (observing the restriction on movement between levels) but cannot change the virtual channel within the same ring. If formulated in the terminology of “nodal cycles” [26], the cycles will be broken at the zero node (for packets moving clockwise) and at the node opposite to it (for packets moving in the other direction). The guaranteed break of the “nodal cycle” is explained by the fact that none of the packets will cross the boundary, which is a consequence of the restriction on the change of the virtual channel and the maximum number of movements around the ring equal to ⌊N/2⌋. For example, based on Figure 3c, the packets entering nodes 0–4 and moving clockwise will never go from the 9-th node to the 0-th since, moving clockwise, the 9-th node can only be reached from the 4-th without exceeding the limit on the number of movements.

## 6. Application of Acyclic Subnetwork to Resolve Deadlocks

A more universal solution, which allows the organization of deadlock-free store-and-forward routing in any topology, is to organize an acyclic subnetwork. The main idea of this approach is described in [13]. The essence of the approach is that any packet that has fallen into a deadlock situation can be sent to the subnetwork to bypass the blocked section. The simplest way to use an acyclic subnetwork is to use a zero-rooted spanning tree. There are two options for organizing routing in a subnetwork.

**Figure 4:**
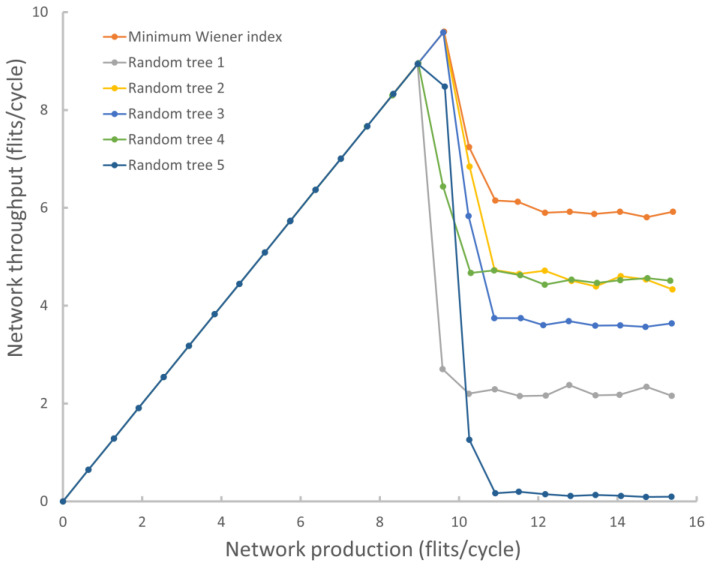
Comparison of different trees as a subnetwork to bypass deadlocks.

Forwarding packets to the subnetwork once guarantees no livelocks but results in very low throughput. Allowing packets to be redirected multiple times to a subnetwork with the ability to exit it can cause the packet to circulate around the network, but this method reduces the load on the subnetwork and gives good throughput of the entire network. Let’s call this method the Acyclic Subnetwork Method (ASM). At the same time, as a result of experiments, it was shown that the characteristics of the network strongly depend on which spanning tree to choose. For example, Figure 4 shows the results of modeling the throughput of a network with an 8 × 8 mesh topology and a standard XY algorithm using various trees as a subnetwork.

The choice of a tree with the minimum routing cost (minimum Wiener index [44]) results in a good throughput, but sometimes (we have not yet been able to determine the regularity), there may be variants of non-Wiener index trees that perform better throughput. Thus, the question of finding the optimal spanning tree for the subnetwork remains open. In addition, the acyclic subnetwork approach [13] does not guarantee the optimality of packet routes, but it does not lead to livelocks [45]. It is also necessary to consider the additional cost of implementing non-standard routers and spanning tree links.

The subnetwork can also be organized using virtual channels, which will reduce hardware costs. Since the underlying topology is the main contributor to the throughput and the subnetwork only resolves deadlocks, moving the subnetwork from physical to virtual links does not greatly affect the overall peak throughput.

**Figure 5:**
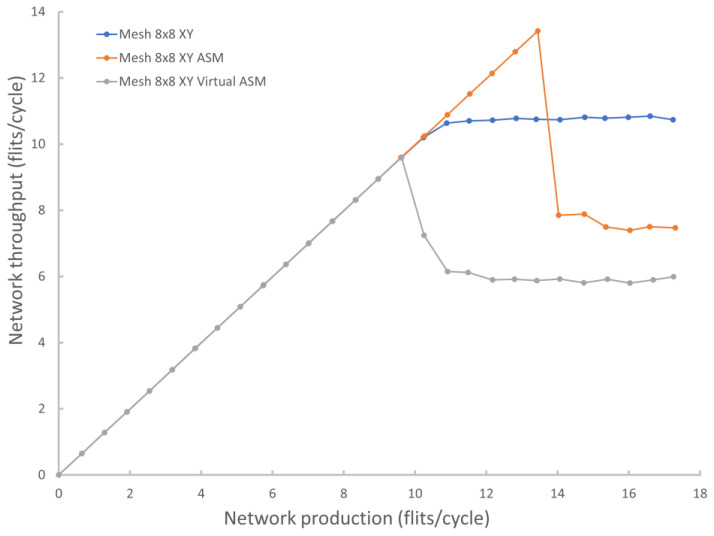
Comparison of throughput of virtual and physical subnetworks when using ASM.

The simulation results of the proposed universal approach with options for an acyclic subnetwork organized with real and virtual channels are shown in Figure 5 (the comparison is made by throughput because this is the most demonstrative parameter characterizing, in general, the capabilities of the network communication subsystem and packet latency). The NoC with a virtual subnetwork has lower throughput than the NoC with a mesh topology and standard XY algorithm. This suggests that the usage of this method of bypassing packet blocking makes sense in the topologies for which a solution that directly takes into account their features has not yet been found. At the same time, the use of a different subnetwork topology or the choice of the optimal spanning tree can significantly improve the peak network throughput.

An important feature of the considered approach is a strong drop in throughput after reaching a certain peak value. This happens due to the fact that at the moment after the peak, the load on the network, created by emerging deadlocks, exceeds the subnetwork throughput. As a result, the overall throughput is reduced almost to the level of the throughput of the subnetwork itself. Thus, it is necessary to control the overall load on the network in order to use it as efficiently as possible. Also, this method does not allow the occurrence of packet tails that block data transfer and, therefore, is applicable only for store-and-forward routing.

## 7. Modeling Routing Methods Using the Ring-Split Algorithm

To analyze the effectiveness of the developed RS algorithm, we compared the network throughput that can be achieved using it with the one that can be obtained in the networks with a mesh topology using the XY algorithm (the comparison is made with flat 2D mesh topology since the circulant is also usually represented in two-dimensional space; XY algorithm is used as a reference one, the most common in NoCs [25]). The Pair Exchange Algorithm [17] was chosen as the routing algorithm in circulants.

**Figure 6:**
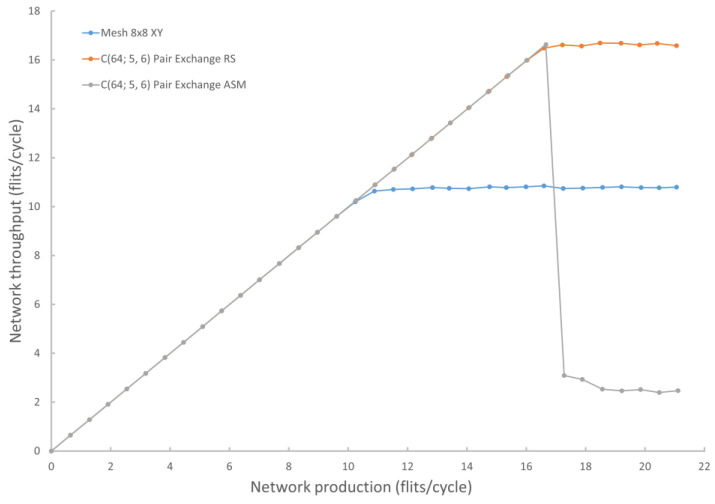
Diagram of throughput for various implementations of a network of 64 nodes using RS, ASM, XY.

With a buffer size of two packets (packet size—five flits of 16 bits each) and store-and-forward routing, the optimal circulant C(64; 5, 6) showed a throughput increase of more than 59% compared to the mesh 8 × 8 topology (Figure 6). The peak throughput, when using the physical implementation of ASM, turned out to be comparable to RS. At the same time, ASM requires two times more communication channels and more complex routers, while RS is just a system of rules which is superimposed on the basic routing algorithm and practically does not require additional overhead.

Since for circulant topologies with an increase in the number of nodes, the diameter increases more slowly than for mesh topologies, for a larger number of nodes, the increase in throughput will be even more noticeable.

As circulants, we used the optimal graphs of the family C(N; D,D+1) (whose properties were considered in [7]) since the considered Pair Exchange Algorithm [17] is applicable to them. With an increase in the number of nodes, the significant advantage of circulant topologies over mesh topologies becomes more and more evident [8]. The 2D-torus topology has a similar diameter to the circulant because it is a more connected graph than the mesh. As is known, some variants of 2D-torus are a special case of non-optimal circulants, and, therefore, the developed RS algorithm can also be applied to them.

## 8. Further Development of the Ring-Split Algorithm

The simulation results show the high prospects of the developed routing method. But they are obtained using the Newxim high-level simulator. This means that they do not take into account the hardware costs for the implementation of the algorithm, the evaluation of which is a separate complex task [46]. In addition, low-level simulation can help to more accurately evaluate the characteristics of the RS algorithm. It is also necessary to analyze the influence of routing algorithms on the average packet latency, which, among other things, depends on both the throughput and the basic algorithm, as well as the characteristics of the NoC topology itself. So, for example, 59% better throughput of C(64; 5, 6) compared to the mesh 8 × 8 topology (Figure 6) is largely explained by the better diameter (six vs. 14), average distance (3.7 vs. 5.3), and bisection width (24 vs. eight) of the circulant.

This work does not investigate how the proposed algorithms behave under conditions of nonuniform distribution of network load, for example, in the presence of hotspots [47,48]. Also, modeling with realistic traffic patterns [26,49] to reflect various modes of network operation is required. These are the tasks for a separate extensive study in the future.

Another promising direction for the development of the proposed RS algorithm is based on the plane tessellation of circulant graphs based on the Cartesian product. According to [50], bipartite circulant graphs can be decomposed into simpler graphs. If there are no cycles in these subgraphs, the rule as follows can be formulated: the packet must change its level (virtual channel) when moving between subgraphs. Under this condition, the cycles in the graph itself will be broken. Network nodes can store the labels of those belonging to their subgraph, and packets can store the labels of the nodes from which they were sent. This will allow us to determine when a packet crosses the boundaries of two subgraphs formed by the plane tessellation. The hypothesis proposed needs testing. If it is correct, the same approach can potentially be applied to other graphs.

## 9. Conclusions

The proposed RS algorithm has a comparable lower peak throughput than the method using a physical ASM in the form of a spanning tree with a minimum Wiener index, and its implementation is much simpler and does not require non-standard routers. In addition, the RS algorithm allows using the wormhole routing if necessary. The developed new approach to deadlock prevention opens up the possibility of the usage of circulants as the topological basis of the NoC using any of the available routing algorithms. As a result of high-level modeling, it was shown that an increase of up to 59% in network throughput is achieved using a new approach for routing in circulants compared to mesh topology with a uniform distribution of network load.

The second described method of routing with bypassing deadlocks by organizing an acyclic subnetwork is universal, but, due to the complexity of implementation, it is worse than the algorithms that take into account the topology features and, therefore, is relevant only for network topologies for which no formalized way to bypass deadlock situations has been found.

## Data Availability

Not applicable.
